# Increased mobilisation of circulating endothelial progenitors in von Hippel-Lindau disease and renal cell carcinoma

**DOI:** 10.1038/bjc.2011.186

**Published:** 2011-06-14

**Authors:** R S Bhatt, A J Zurita, A O'Neill, A Norden-Zfoni, L Zhang, H K Wu, P Y Wen, D George, V P Sukhatme, M B Atkins, J V Heymach

**Affiliations:** 1 Division of Hematology-Oncology and Cancer Biology, Beth Israel Deaconess Medical Center, 375 Longwood Avenue, MASCO 426, Boston, MA 02215, USA; 2 Genitourinary Medical Oncology, The University of Texas MD Anderson Cancer Center, Houston, TX 77030, USA; 3 Department of Biostatistics and Computational Biology, Dana-Farber Cancer Institute; Harvard Medical School, Children’s Hospital, Boston, MA 02215, USA; 4 Departments of Thoracic/Head and Neck Medical Oncology and Cancer Biology, Division of Cancer Medicine, The University of Texas MD Anderson Cancer Center, Houston, TX 77030, USA; 5 Center for Neuro-Oncology, Dana-Farber/Brigham and Women’s Cancer Center, Boston, MA 02115, USA; 6 Divisions of Medical Oncology and Urology, Duke University Medical Center, Durham, NC 27705, USA; 7 Division of Interdisciplinary Medicine and Biotechnology, Department of Medicine, Beth Israel Deaconess Medical Center, Boston, MA 02215, USA

**Keywords:** circulating endothelial cells, von Hippel-Lindau (VHL) disease, renal cancer, biomarker, angiogenesis

## Abstract

**Background:**

Circulating endothelial cells (CECs) are a candidate biomarker for monitoring angiogenesis in cancer. Circulating endothelial cell subsets are mobilised by angiogenic mediators. Because of the highly angiogenic phenotype of renal cell carcinoma (RCC), we sought to assess the potential of CECs as a marker of RCC in patients with von Hippel-Lindau (VHL) disease and those with sporadic RCC.

**Methods:**

We performed multicolour flow cytometry to enumerate CECs in patients with RCC, patients with VHL disease with and without RCC, and normal subjects. Two subsets of CECs were evaluated: mature CECs (mCECs) and circulating endothelial progenitors (CEPs).

**Results:**

In patients with VHL disease and RCC and those with sporadic RCC (*N*=10), CEPs and the CEP:mCEC ratio were higher than in normal subjects (*N*=17) (median CEPs: 0.97 *vs* 0.19 cells μl^−1^, respectively, *P*<0.01; median CEP:mCEC: 0.92 *vs* 0.58, respectively, *P*=0.04). However, in patients with VHL without RCC, CECs were not increased. In paired pre- and post-nephrectomy RCC patient samples (*N*=20), CEPs decreased after surgery (median difference 0.02 cells μl^−1^, −0.06 to 1.2; *P*=0.05).

**Conclusion:**

Circulating endothelial progenitors were elevated in RCC, but not in patients with VHL without RCC. Circulating endothelial progenitor enumeration merits further investigation as a monitoring strategy for patients with VHL.

Renal cell carcinomas (RCCs) are the most frequent malignant tumours to occur in patients with von Hippel-Lindau (VHL) disease, and VHL disease is the primary cause of inherited renal cancer. von Hippel-Lindau disease affected individuals are at risk for the development of up to 600 clear cell renal carcinomas per kidney (Kaelin, 2008). von Hippel-Lindau disease arises from heterozygous germline mutations in the *VHL* tumour suppressor gene, which resides on chromosome 3p25, and it is characterised by clear cell RCC, hemangiomas, pheochromocytomas, and other tumour types (Linehan *et al*, 2003). The development of tumours in VHL disease results from loss or inactivation of the remaining wild-type allele, leading to an absence of functional VHL protein. Somatic *VHL* mutations are also common in sporadic clear cell RCC and hemangioblastomas (Linehan *et al*, 2003). Restoration of VHL protein function is sufficient to suppress tumour formation *in vivo* in VHL-defective renal carcinoma cells (Linehan *et al*, 2003).

The *VHL* gene encodes an E3 ligase involved in the oxygen-dependent ubiquitination and proteasomal degradation of the hypoxia-inducible factors HIF-1α and HIF-2α, subunits of transcriptional factors involved in the expression of vascular endothelial growth factor (VEGF) and other hypoxia-driven genes. The loss of VHL protein results in the accumulation of HIF (even in normoxic conditions) and the overproduction of VEGF and other mediators of angiogenesis (Linehan *et al*, 2003). As a result, RCCs are highly vascular tumours and are responsive to therapy with agents that interfere with VEGF signalling.

RCCs often remain asymptomatic for long intervals before diagnosis. In patients with VHL, who also develop RCC, the frequent absence of clinical symptoms underscores the importance of early detection, as diagnosis during presymptomatic screening has the potential to enhance overall outcomes. Although serial imaging of the kidneys is useful in this regard, more effective strategies for monitoring patients with VHL disease are needed. Also, new biomarkers for disease recurrence after nephrectomy in patients with sporadic RCC could indicate the early need for intervention.

Circulating endothelial cells (CECs) are a candidate biomarker for monitoring angiogenesis in patients with VHL disease and in those with RCC. Circulating endothelial cells are known to express VEGF receptors and to be mobilised by VEGF in patients and murine models, and they have been found at increased levels in cancer patients (Asahara *et al*, 1997, 1999; Kalka *et al*, 2000; Peichev *et al*, 2000; Mancuso *et al*, 2001; Schuch *et al*, 2003; Ceradini *et al*, 2004) including RCC (Gruenwald *et al*, 2010). Two main types of CECs have been described: mature CECs (mCECs), which are thought to be shed from preexisting blood vessels, and bone marrow-derived circulating endothelial progenitors (CEPs). Circulating endothelial progenitor mobilisation has been shown to depend on HIF-regulated factors such as VEGF and SDF-1α. Furthermore, mCECs and CEPs may respond differently to angiogenic stimuli and provide distinct information regarding angiogenic drive and response to treatment (Bertolini *et al*, 2003; Beaudry *et al*, 2005; Shaked *et al*, 2005; Batchelor *et al*, 2007). Thus, because of the highly angiogenic phenotype of RCC and its dependence on HIF and HIF-regulated factors, we sought to assess the potential of using mCECs and CEPs as potential biomarkers of RCC in the VHL patient population and also in patients with sporadic RCC. We hypothesised that CEPs, and the ratio of CEPs to mCECs, would be altered in RCC patients but not in VHL patients without RCC.

## Materials and methods

### Patient population

Patients and healthy control subjects, whose disease status was assessed radiologically, were enroled in IRB-approved protocols at Beth Israel Deaconess Medical Center (Boston, MA, USA) and The University of Texas MD Anderson Cancer Center (Houston, TX, USA). Patients with sporadic RCC and those with VHL–RCC were covered by a Dana-Farber–Harvard Cancer Center tissue-collection protocol and an MD Anderson laboratory-collection protocol that enabled blood sampling before and after nephrectomy.

For the purposes of this study, we divided those trial participants into various cohorts as follows: (a) Patients with VHL disease (*N*=13), including those with no radiologic evidence of RCC but with benign tumours in the brain, eye, kidney, spinal cord, or pancreas, or a history of resected RCC (*N*=7); and additional patients with VHL and active RCC (VHL–RCC; *N*=6); (b) Patients with sporadic metastatic RCC (*N*=4;); (c) Individual patients with non-metastatic sporadic RCC (*N*=20) who underwent nephrectomy; paired blood samples were obtained within 4 weeks before nephrectomy and 6 weeks to 6 months after nephrectomy; (d) Healthy control subjects (*N*=17); and (e) Additional healthy control subjects (*N*=18) and patients with sporadic metastatic RCC (*N*=9) as an independent validation cohort for CEPs.

### Blood collection

Peripheral blood of patients was collected in Vacutainer CPT tubes with citrate (BD Biosciences, Franklin Lakes, NJ, USA) and spun to separate the PBMC layer as previously described (Kalka *et al*, 2000; Peichev *et al*, 2000; Norden-Zfoni *et al*, 2007). The PBMCs were frozen and stored in liquid nitrogen until the day of analysis.

### Flow cytometry

Batch analysis of PBMCs was performed to minimise interassay variability. Circulating endothelial cells were enumerated by using four-colour flow cytometry as described previously (Beaudry *et al*, 2005; Norden-Zfoni *et al*, 2007; Mancuso *et al*, 2009). Briefly, cells were washed with PBS with 1% albumin and incubated with a panel of antibodies to establish CEC phenotype, including anti-CD45, -CD31, -CD146, and -CD133 (BD Biosciences, San Jose, CA, USA). The mCECs were defined as staining negatively for the haematopoietic marker CD45, positively for the endothelial markers CD31 and CD146, and negatively for the progenitor marker CD133. Circulating endothelial progenitors had the same phenotype except they were positive for CD133. Human umbilical vein endothelial cells (Cambrex, East Rutherford, NJ, USA) were used as positive controls for endothelial marker staining, and WERI cells (American Type Culture Collection, Manassas, VA, USA) were used as positive controls for CD133. CD146 was not available for the validation set, and only CEPs were determined. The percentages of stained cells were determined by comparison with the appropriate fluorescent isotype controls.

To establish absolute CEC counts, the volume of blood analyzed was determined by using the lymphocyte or monocyte counts obtained from the patients’ differential.

### Statistical analysis

Exact Wilcoxon rank-sum testing was used to compare CEP and mCEC counts and mCEP:CEC ratios between groups. For the 20 patients who underwent nephrectomy, Wilcoxon signed-rank test was used to look at changes in those values in terms of absolute differences between the samples collected before and after nephrectomy.

## Results

### CEP and mCEC counts are higher in RCC patients than in healthy control subjects

**Figure 1:**
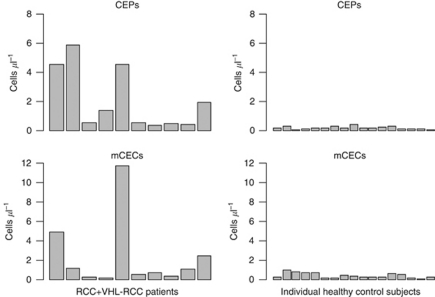
Graphs show the absolute numbers of CEP and mCEC cells (per microlitre) in individual RCC patients (*N*=10, 4 with sporadic non-metastatic RCC and 6 with VHL–RCC) and healthy control subjects (*N*=17). Number of cells varied widely within the RCC patient cohort.

**Figure 2:**
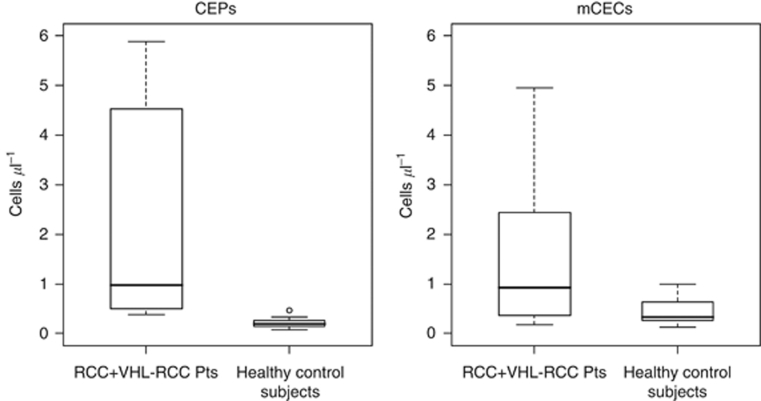
Box plots demonstrate that RCC patients have more CEPs and mCECs than healthy control subjects have. Data (medians and ranges) are from 10 patients with RCC (4 with sporadic non-metastatic RCC and 6 with VHL–RCC) and 17 healthy control subjects. The thick horizontal line within each box is the median value; the upper and lower boundaries of the boxes are the 75th and 25th percentiles. The bars above and below the boxes are placed at the observed values closest to the 75th percentile plus 1.5 times the interquartile range (75th percentile minus 25th percentile) and closest to the 25th percentile minus 1.5 times the interquartile range; observations outside those limits are plotted separately and represented with a small circle. Observations more extreme than 6 were excluded from these figures.

PBMC fractions from patients with RCC and healthy control subjects were analyzed for CEP and mCEC counts by using multiparametric flow cytometry (Norden-Zfoni *et al*, 2007; Mancuso *et al*, 2009). As shown in Figure 1, the absolute numbers of CEPs and mCECs for individual patients were higher in those with RCC (*N*=10, four with sporadic RCC and six with VHL–RCC) than they were in the healthy control subjects (*N*=17), with wide variability within the RCC cohort. In RCC patients *vs* healthy control subjects, the median numbers (range) of CEPs and mCECs were 0.97 cells μl^−1^ (0.39–5.88) *vs* 0.19 cells μl^−1^ (0.08–0.47; *P*<0.01) and 0.93 cells μl^−1^ (0.19–11.75) *vs* 0.33 cells μl^−1^ (0.12–0.99; *P*=0.05), respectively (Figure 2).

Similar differences were seen in the independent CEP validation cohort: the median number (range) of CEPs in RCC patients (*N*=9) was 1.4 cells μl^−1^ (0.09–11.99) *vs* 0.40 cells μl^−1^ (0.17–1.89) in healthy control subjects (*N*=18; *P*<0.05).

### CEP:mCEC ratios are higher in RCC patients than in healthy control subjects

**Figure 3:**
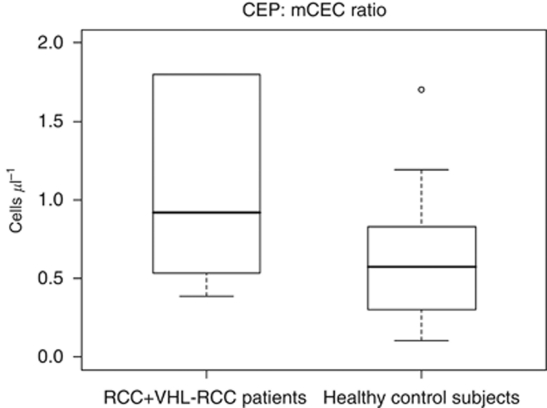
Box plot shows that RCC patients also have a higher CEP:mCEC ratio than healthy control subjects have. Plot is read as described in Figure 2. Observations more extreme than 2 were excluded from these figures.

Previous studies have shown that various anti-cancer therapies can have different effects on CEPs and mCECs. For example, some VEGF inhibitors have been shown to increase mCECs, whereas decreasing CEPs in preclinical models (Beaudry *et al*, 2005). To investigate this further, we evaluated the CEP:mCEC ratios in RCC patients and healthy control subjects. We found that RCC patients (*N*=10) had a median CEP:mCEC ratio of 0.92 (range 0.39–7.52), which was greater than that in the control subjects (*N*=17; median 0.58, range 0.10–1.70; *P*<0.05; Figure 3).

### CEP counts and CEP:mCEC ratios are higher in VHL–RCC patients than in VHL patients without RCC

**Figure 4:**
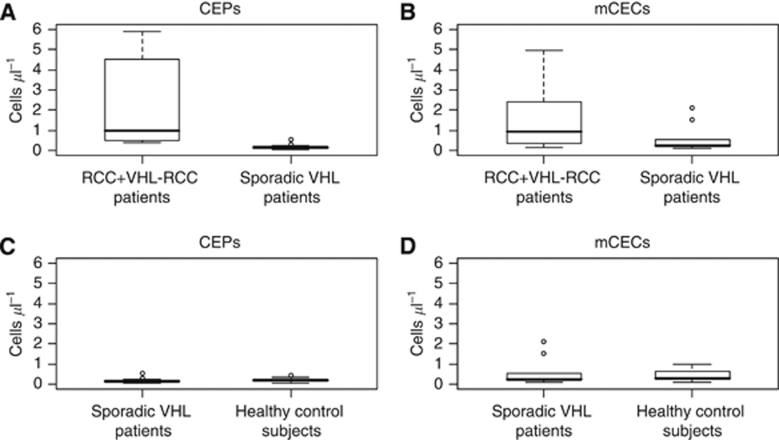
Box plots demonstrate that the cohort of patients with sporadic RCC plus those with VHL–RCC (total *N*=10) has more CEPs (**A**) and mCECs (**B**) than the VHL patients with no RCC (*N*=13) have. As **C** and **D** show, no differences in CEP and mCEC counts were found between the cohort of patients with VHL without RCC (*N*=13) and the cohort of healthy control subjects (*N*=17). Plots are read as described in Figure 2.

When compared with the patients with sporadic RCC plus those with VHL–RCC (total *N*=10), the patients with VHL but no RCC (*N*=13) had fewer CEPs, with a median (range) of 0.15 cells μl^−1^ (0.07–0.55) *vs* 0.97 cells μl^−1^ (0.39–5.88) (*P*<0.01) and also fewer mCECs, with 0.26 cells μl^−1^ (0.11–2.11) *vs* 0.93 cells μl^−1^ (0.19–11.75) (*P*=0.02) (Figures 4A and B).

**Figure 5:**
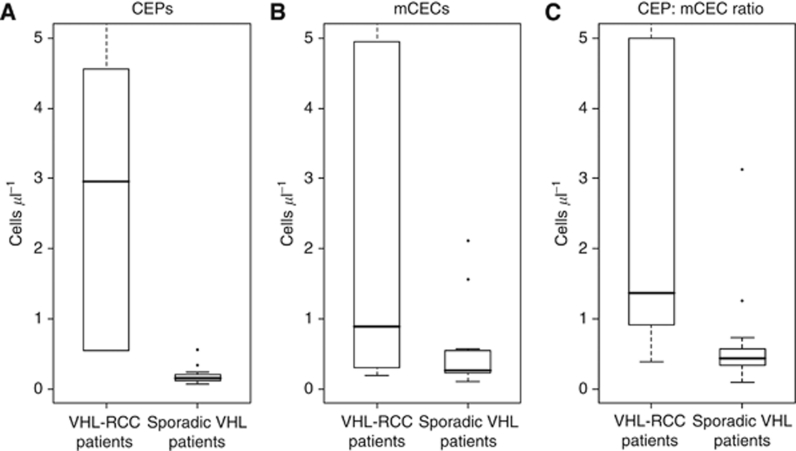
Box plots show that patients with VHL–RCC have more CEPs (**A**) and a higher CEP:mCEC ratio (**C**) than patients with VHL with no RCC have, but no different mCECs (**B**). Plots are read as described in Figure 2. Observations more extreme than 5 were excluded from these figures.

We additionally compared CEP and mCEC counts and CEP:mCEC ratios from the VHL–RCC patients (*N*=6) with those from the patients with VHL and no RCC (*N*=13). Patients with VHL–RCC had more CEPs and higher CEP:mCEC ratios than patients with VHL without RCC (CEPs, 2.96 cells μl^−1^ *vs* 0.15 cells μl^−1^, *P*<0.01; CEP:mCEC, 1.36 *vs* 0.44, *P*=0.02) (Figures 5A and C). The VHL–RCC patients also had more mCECs than the VHL patients without RCC had, but the difference was not statistically significant (0.89 *vs* 0.26 cells μl^−1^; *P*=0.11) (Figure 5B).

### CEPs and mCEC counts and CEP:mCEC ratios are comparable in VHL patients with no RCC and healthy control subjects

No difference in CEPs and mCEC counts (Figures 4C and D) and CEP:mCEC ratios were found between the VHL patients with no RCC (*N*=13) and the healthy control subjects (*N*=17; all *P* values >0.05).

### CEP counts decrease after nephrectomy in patients with non-metastatic sporadic RCC

To further test whether CEP counts correlate with the presence of RCC, we studied paired PBMC specimens from a separate cohort of patients with sporadic RCC before and after nephrectomy (*N*=20). These patients had RCC confined to the kidney and no evidence of metastatic disease. In this patient cohort, the CEP counts decreased after nephrectomy: the median change (range) between before and after nephrectomy was 0.02 cells μl^−1^ (−0.06 to 1.2, *P*=0.05). In contrast, the mCEC counts showed large variability, and the CEP:mCEC ratio did not change significantly between before and after nephrectomy (Table 1).

## Discussion

von Hippel-Lindau disease predisposes patients to clear cell RCC, a highly vascular tumour type that frequently involves activation of the HIF pathway, an established regulator of VEGF and other angiogenic cytokines. Given that several of these factors are known to increase the numbers of mCECs and CEPs in the circulation of human subjects (Kalka *et al*, 2000; Beaudry *et al*, 2005; Farace *et al*, 2007), we compared the CEC counts in healthy control subjects with those in patients with VHL with and without RCC and in patients with sporadic RCC. We found in two independent cohorts that RCC patients have more CEPs (and higher CEP:mCEC ratios) than healthy control subjects do and that in RCC patients, the CEP count decreases after surgical removal of the primary tumour (i.e., nephrectomy). Although it was not the primary focus of this study, it is also worth noting that patients with VHL plus tumours other than RCC have fewer CEPs and lower CEP:mCEC ratios than patients with VHL–RCC have, suggesting that RCC may promote CEP increase to a greater extent than other tumours in VHL patients.

These findings are consistent with the biology of VHL disease, in which HIF levels and VEGF signalling are increased (Ohh *et al*, 2000; Rini and Small, 2005). von Hippel-Lindau itself in the absence of RCC did not lead to significant differences in overall CEC numbers, suggesting that the development of renal cancer, and not merely the presence of *VHL* mutation, is associated with the increase in CEPs. Possibly, this observation reflects the greater production of VEGF or other factors known to promote the survival and/or mobilisation of CEPs and other bone marrow-derived cells by renal cancer tissue, although the specific factor(s) contributing to the relatively elevated levels of CEPs in this study are not known.

Thus, it is possible that counting the CEC subsets, particularly the CEPs, would be a means of surveillance of VHL patients at risk for developing RCC. Moreover, given that patients with stage IV RCC have more CECs than are present after nephrectomy for localised disease, CECs could also be useful as biomarkers for detecting early disease recurrence in patients who have undergone such nephrectomy. The results of more detailed studies to relate disease site and tumour burden with CEC counts may provide further support for the use of CEC analysis in RCC surveillance.

Patients with RCC also had a higher CEP:mCEC ratio than both the healthy control subjects and the VHL patients without RCC had. We propose that the CEP:mCEC ratio may serve as a novel biomarker reflecting the balance of angiogenic drive of the host as reflected in the blood. Indeed, in both preclinical models and phase I/II clinical trials, treatment with antiangiogenic agents has been shown to inhibit CEP mobilisation (Daenen *et al*, 2009) and exert different effects on CEPs and mCECs (Beaudry *et al*, 2005). Moreover, antiangiogenic therapy induces dynamic changes in CEPs and mCECs that may correlate with clinical benefit: earlier studies conducted by our group and others found CECs useful as a surrogate marker for antiangiogenic therapy (Beaudry *et al*, 2005; Shaked *et al*, 2005; Norden-Zfoni *et al*, 2007; Calleri *et al*, 2009; Willett *et al*, 2009), as an aid in selecting optimal dosing (Shaked *et al*, 2005), and in assessing benefit to patients (Norden-Zfoni *et al*, 2007; Vroling *et al*, 2009). For example, specific changes in CEC counts or CEP:mCEC ratio may be useful as a marker for predicting a patient's prognosis or response to therapy with VEGF receptor inhibitors.

Studies to relate CEC counts with the effects of therapy with VEGF receptor inhibitors in RCC patients are under way, but common problems have been the different criteria used for defining CECs and the difficulty in accurately counting these rare cells (Bhatt *et al*, 2007; Calleri *et al*, 2009; Mancuso *et al*, 2009). As CEC counts vary widely within the population of patients with RCC, serial measurements in individual patients may prove especially interesting. Additionally, RCC patients with different cytokine profiles and CEC responses may require different management. Studies with larger numbers of RCC patients treated with angiogenesis inhibitors and other agents will be required to resolve these questions.

## Figures and Tables

**Table 1:** Changes in CECs in paired blood specimens obtained before and after nephrectomy in 20 patients with non-metastatic sporadic RCC

**CEC factor**	**Median difference (range) from ‘before nephrectomy’ value**	***P* a**
CEP count (cells μl^−1^)	0.02 (−0.06 to 1.2)	0.05
mCEC count (cells μl^−1^)	−0.08 (−52.56 to 32)	0.75
CEP:mCEC ratio	0.001 (−0.05 to 0.037)	0.20

Abbreviations: CECs=circulating endothelial cells; RCC=renal cell carcinoma; CEP=circulating endothelial progenitor; mCEC=mature CEC.

a Wilcoxon signed-rank test.

## References

[bib1] Asahara T, Murohara T, Sullivan A, Silver M, van der Zee R, Li T, Witzenbichler B, Schatteman G, Isner JM (1997) Isolation of putative progenitor endothelial cells for angiogenesis. Science 275: 964–967 9020076 10.1126/science.275.5302.964

[bib2] Asahara T, Takahashi T, Masuda H, Kalka C, Chen D, Iwaguro H, Inai Y, Silver M, Isner JM (1999) VEGF contributes to postnatal neovascularization by mobilizing bone marrow-derived endothelial progenitor cells. EMBO J 18: 3964–3972 10406801 10.1093/emboj/18.14.3964 PMC1171472

[bib3] Batchelor TT, Sorensen AG, di Tomaso E, Zhang WT, Duda DG, Cohen KS, Kozak KR, Cahill DP, Chen PJ, Zhu M, Ancukiewicz M, Mrugala MM, Plotkin S, Drappatz J, Louis DN, Ivy P, Scadden DT, Benner T, Loeffler JS, Wen PY, Jain RK (2007) AZD2171, a pan-VEGF receptor tyrosine kinase inhibitor, normalizes tumor vasculature and alleviates edema in glioblastoma patients. Cancer Cell 11: 83–95 17222792 10.1016/j.ccr.2006.11.021 PMC2748664

[bib4] Beaudry P, Force J, Naumov GN, Wang A, Baker CH, Ryan A, Soker S, Johnson BE, Folkman J, Heymach JV (2005) Differential effects of vascular endothelial growth factor receptor-2 inhibitor ZD6474 on circulating endothelial progenitors and mature circulating endothelial cells: implications for use as a surrogate marker of antiangiogenic activity. Clin Cancer Res 11: 3514–3522 15867254 10.1158/1078-0432.CCR-04-2271

[bib5] Bertolini F, Paul S, Mancuso P, Monestiroli S, Gobbi A, Shaked Y, Kerbel RS (2003) Maximum tolerable dose and low-dose metronomic chemotherapy have opposite effects on the mobilization and viability of circulating endothelial progenitor cells. Cancer Res 63: 4342–4346 12907602

[bib6] Bhatt RS, Seth P, Sukhatme VP (2007) Biomarkers for monitoring antiangiogenic therapy. Clin Cancer Res 13: 777s–780s 17255309 10.1158/1078-0432.CCR-06-1922

[bib7] Calleri A, Bono A, Bagnardi V, Quarna J, Mancuso P, Rabascio C, Dellapasqua S, Campagnoli E, Shaked Y, Goldhirsch A, Colleoni M, Bertolini F (2009) Predictive potential of angiogenic growth factors and circulating endothelial cells in breast cancer patients receiving metronomic chemotherapy plus bevacizumab. Clin Cancer Res 15: 7652–7657 19996223 10.1158/1078-0432.CCR-09-1493

[bib8] Ceradini DJ, Kulkarni AR, Callaghan MJ, Tepper OM, Bastidas N, Kleinman ME, Capla JM, Galiano RD, Levine JP, Gurtner GC (2004) Progenitor cell trafficking is regulated by hypoxic gradients through HIF-1 induction of SDF-1. Nat Med 10: 858–864 15235597 10.1038/nm1075

[bib9] Daenen LG, Shaked Y, Man S, Xu P, Voest EE, Hoffman RM, Chaplin DJ, Kerbel RS (2009) Low-dose metronomic cyclophosphamide combined with vascular disrupting therapy induces potent antitumor activity in preclinical human tumor xenograft models. Mol Cancer Ther 8: 2872–2883 19825805 10.1158/1535-7163.MCT-09-0583 PMC2782518

[bib10] Farace F, Massard C, Borghi E, Bidart JM, Soria JC (2007) Vascular disrupting therapy-induced mobilization of circulating endothelial progenitor cells. Ann Oncol 18: 1421–1422 17693656 10.1093/annonc/mdm367

[bib11] Gruenwald V, Beutel G, Schuch-Jantsch S, Reuter C, Ivanyi P, Ganser A, Haubitz M (2010) Circulating endothelial cells are an early predictor in renal cell carcinoma for tumor response to sunitinib. BMC Cancer 10: 695 21194438 10.1186/1471-2407-10-695 PMC3023793

[bib12] Kaelin Jr WG (2008) The von Hippel-Lindau tumour suppressor protein: O2 sensing and cancer. Nat Rev Cancer 8: 865–873 18923434 10.1038/nrc2502

[bib13] Kalka C, Masuda H, Takahashi T, Gordon R, Tepper O, Gravereaux E, Pieczek A, Iwaguro H, Hayashi SI, Isner JM, Asahara T (2000) Vascular endothelial growth factor(165) gene transfer augments circulating endothelial progenitor cells in human subjects. Circ Res 86: 1198–1202 10864908 10.1161/01.res.86.12.1198

[bib14] Linehan WM, Walther MM, Zbar B (2003) The genetic basis of cancer of the kidney. J Urol 170: 2163–2172 14634372 10.1097/01.ju.0000096060.92397.ed

[bib15] Mancuso P, Antoniotti P, Quarna J, Calleri A, Rabascio C, Tacchetti C, Braidotti P, Wu HK, Zurita AJ, Saronni L, Cheng JB, Shalinsky DR, Heymach JV, Bertolini F (2009) Validation of a standardized method for enumerating circulating endothelial cells and progenitors: flow cytometry and molecular and ultrastructural analyses. Clin Cancer Res 15: 267–273 19118054 10.1158/1078-0432.CCR-08-0432

[bib16] Mancuso P, Burlini A, Pruneri G, Goldhirsch A, Martinelli G, Bertolini F (2001) Resting and activated endothelial cells are increased in the peripheral blood of cancer patients. Blood 97: 3658–3661 11369666 10.1182/blood.v97.11.3658

[bib17] Norden-Zfoni A, Desai J, Manola J, Beaudry P, Force J, Maki R, Folkman J, Bello C, Baum C, DePrimo SE, Shalinsky DR, Demetri GD, Heymach JV (2007) Blood-based biomarkers of SU11248 activity and clinical outcome in patients with metastatic imatinib-resistant gastrointestinal stromal tumor. Clin Cancer Res 13: 2643–2650 17473195 10.1158/1078-0432.CCR-06-0919

[bib18] Ohh M, Park CW, Ivan M, Hoffman MA, Kim TY, Huang LE, Pavletich N, Chau V, Kaelin WG (2000) Ubiquitination of hypoxia-inducible factor requires direct binding to the beta-domain of the von Hippel-Lindau protein. Nat Cell Biol 2: 23–47 10.1038/35017054 10878807

[bib19] Peichev M, Naiyer AJ, Pereira D, Zhu Z, Lane WJ, Williams M, Oz MC, Hicklin DJ, Witte L, Moore MA, Rafii S (2000) Expression of VEGFR-2 and AC133 by circulating human CD34(+) cells identifies a population of functional endothelial precursors. Blood 95: 952–958 10648408

[bib20] Rini BI, Small EJ (2005) Biology and clinical development of vascular endothelial growth factor-targeted therapy in renal cell carcinoma. J Clin Oncol 23: 1028–1043 15534359 10.1200/JCO.2005.01.186

[bib21] Schuch G, Heymach JV, Nomi M, Machluf M, Force J, Atala A, Eder Jr JP, Folkman J, Soker S (2003) Endostatin inhibits the vascular endothelial growth factor-induced mobilization of endothelial progenitor cells. Cancer Res 63: 8345–8350 14678995

[bib22] Shaked Y, Bertolini F, Man S, Rogers MS, Cervi D, Foutz T, Rawn K, Voskas D, Dumont DJ, Ben-David Y, Lawler J, Henkin J, Huber J, Hicklin DJ, D’Amato RJ, Kerbel RS (2005) Genetic heterogeneity of the vasculogenic phenotype parallels angiogenesis; Implications for cellular surrogate marker analysis of antiangiogenesis. Cancer Cell 7: 101–111 15652753 10.1016/j.ccr.2004.11.023

[bib23] Willett CG, Duda DG, di Tomaso E, Boucher Y, Ancukiewicz M, Sahani DV, Lahdenranta J, Chung DC, Fischman AJ, Lauwers GY, Shellito P, Czito BG, Wong TZ, Paulson E, Poleski M, Vujaskovic Z, Bentley R, Chen HX, Clark JW, Jain RK (2009) Efficacy, safety, and biomarkers of neoadjuvant bevacizumab, radiation therapy, and fluorouracil in rectal cancer: a multidisciplinary phase II study. J Clin Oncol 27: 3020–3026 19470921 10.1200/JCO.2008.21.1771 PMC2702234

[bib24] Vroling L, van der Veldt AA, de Haas RR, Haanen JB, Schuurhuis GJ, Kuik DJ, van Cruijsen H, Verheul HM, van den Eertwegh AJ, Hoekman K, Boven E, van Hinsbergh VW, Broxterman HJ (2009) Increased numbers of small circulating endothelial cells in renal cell cancer patients treated with sunitinib. Angiogenesis 12: 69–79 19212818 10.1007/s10456-009-9133-9

